# Addis Ababa population-based pattern of cancer therapy, Ethiopia

**DOI:** 10.1371/journal.pone.0219519

**Published:** 2019-09-19

**Authors:** Jana Feuchtner, Assefa Mathewos, Asmare Solomon, Genebo Timotewos, Abreha Aynalem, Tigeneh Wondemagegnehu, Amha Gebremedhin, Fekadu Adugna, Mirko Griesel, Andreas Wienke, Adamu Addissie, Ahmedin Jemal, Eva Johanna Kantelhardt

**Affiliations:** 1 Institute of Medical Epidemiology, Biostatistics and Informatics, Martin-Luther-University Halle-Wittenberg, Halle, Germany; 2 Radiotherapy Center, Addis Ababa University, Addis Ababa, Ethiopia; 3 Division of Hematology, Department of Internal Medicine, Addis Ababa University, Addis Ababa, Ethiopia; 4 School of Public Health, Addis Ababa University, Addis Ababa, Ethiopia; 5 Surveillance and Health Service Reseach, American Cancer Society, Atlanta, Georgia, United States of America; 6 Department of Gynaecology, Medical Faculty, Martin-Luther-University Halle-Wittenberg, Halle, Germany; Kaiser Permanente Washington Health Research Institute, UNITED STATES

## Abstract

Cancer in Sub-Saharan Africa is becoming an important challenge for health services due to rising numbers of patients. In Addis Ababa with around 3.5 million inhabitants, more than 2000 cases are diagnosed annually. In this retrospective population-based cohort study we assessed completeness of and waiting time for cancer-therapy among patients registered in the Addis Ababa City Cancer Registry (AACCR), Ethiopia. Patient hospital files were retrieved to complete the data from AACCR. A total of 588 files were found (51% of those diagnosed from January to March 2012 and 2014). We analyzed completeness and waiting time of chemotherapy and radiotherapy; with completeness defined as ≥85% therapy received according to local guidelines. Analysis was done for the five most common cancer-types commonly treated with chemotherapy (breast, colorectal, non-Hodgkin`s lymphoma, lung and ovarian) and the four most common cancer-types commonly treated with radiotherapy (breast, cervical, head and neck and rectal). In our study, half of the patients (54.1%) received adequately dosed chemotherapy and 24.5% of patients received adequately dosed radiotherapy. The median waiting time was 2.1 months (Range: 0 to 20.72) for chemotherapy and 7 months (Range: 0.17 to 21.8) for radiotherapy. This study underscores the need for health system measures to improve cancer-directed therapy in Ethiopia, especially concerning radiotherapy.

## Introduction

Cancer in sub-Saharan Africa (SSA) is on the rise caused by a rapid population growth, higher life expectancy and adoption of unhealthy lifestyles [1], [2]. Africa`s population is growing rapidly. According to UN estimates, the continent will double from 1.2 billion people in 2015 to 2.5 billion in 2050 [3], making its population the fastest growing worldwide with a shift towards an older age distribution [4]. This makes cancer a severe force to be reckoned with and a huge challenge for the health care systems of Africa.

Cancer therapy options in most SSA countries are sparse and when available, they are unable to sufficiently serve patients`needs [5]. The Concord 2 study has shown that cancer survival rates differ significantly around the world, with Africa in last place for most types of cancer [6]. This striking fact is most likely due to late-stage presentation [7] and poor access to therapy [5]. Studies estimate that 80% of the 15.2 million new cancer cases in 2015 will need a surgical intervention at least once. Yet, only 25% of cancer patients worldwide and less than 5% in low-income countries get timely, affordable and safe surgery [8]. Cancer diagnoses in some SSA countries are often solely based on a clinical diagnosis not verified through biopsy, making cancer care an even more difficult task [9]. The use of adjuvant therapy in SSA has steadily increased in the past decades. Surgeons used to be the ones responsible for chemotherapy, but there has been a rise in the number of oncologists in the past years [10]- however still not enough to serve the increasing demand. The main obstacle to sufficient cancer chemotherapy is the availability and cost of chemotherapeutic agents. The use of generic drugs from Asia is common; patented drugs are often not affordable which can lead to chemo-morbidity due to different bio-equivalencies and efficacies [10]. Only 23 out of 52 countries in Africa have radiotherapy available, Southern and Northern Africa possessing 90% of the total machines available [11]. In this study, we aimed to describe pattern of therapy of individual cancer patients from Addis Ababa, Ethiopia. Roughly 81% of the 107 million Ethiopian population lives in rural areas, 3.5 million in Addis Ababa [12], [13]. There were 0.03 physicians for every 1000 people in 2016 compared to 3.7 in high-income European countries [14]. The Tikur Anbessa specialized hospital was Ethiopia`s only center for cancer offering oncologic surgery, chemotherapy and radiotherapy with one cobalt-60 teletherapy machine. The hospital had a capacity of 600 beds; 18 beds were dedicated to cancer patients [15]. A study from 2006 estimated a demand of 85 additional radiotherapy-machines for Ethiopia and highlighted the tremendous health service deficit [16]. A total of just over 2000 new cancer cases were detected annually in the Addis Ababa population-based cancer registry (AACCR) [17], which was founded 2011. The AACCR data is the basis for the WHO Globocan estimations [18].

Little is known about pattern of cancer therapy in settings with limited resources such as Ethiopia. This study aims to provide an overview of cancer stages and therapy using individual patient data from the AACCR. A cohort from 2012 (longer follow-up, assumed more difficult to access) and a second cohort from 2014 (shorter follow-up, assumed easier to access) were chosen for data collection at the end of 2015 to assess feasibility of obtaining sufficient details of information. This data will be the basis to assess the unmet need for cancer treatment in Addis Ababa, Ethiopia.

## Methods and materials

### Study design

This retrospective population-based cohort study was conducted within the population-based AACCR.

### Setting, participants and variables

AACCR actively collects all new cancer patients who are residents of Addis Ababa from 20 collaborating institutions (pathology, oncology and radiotherapy facilities). Basic information is documented; due to time constraints, details about therapy are not registered. All cancer patients registered in the AACCR between January 1^st^ and March 31^st^ of the years 2012 and 2014 were included in this study, thus assuming a random sample. Hospital files of the registered patients were retrieved between October 2015 and February 2016 to complete information on therapy.

### Study size and bias

The original sample consisted of 1149 patients, registered by the AACCR. The tracing rate of the hospital files in the 1012 cohort was 48.4% and 62.4% in the 2014 cohort. A total of 44 patients had to be excluded due to primarily false registration (e.g. benign disease). The resulting study size consisted of 588 patients with information from files available (51.2% of the 1149 AACCR cases); the remaining 48.8% files were not retrieved.

To investigate selection bias of the study population, proportions of known characteristics were compared between the AACCR cohort and the study cohort.

We expected files from 2012 would possibly be more difficult to obtain compared to more recent files from 2014. In case the proportion of files detected as well as completeness of therapy did not differ much between the 2012 and 2014 cohort, we planned to combine both for analysis.

### Staging

Tumors were classified according to the International Union for Cancer Control (UICC) [19] and assessed at time-point of diagnosis. Gynecologic tumors were staged according to the International Federation of Gynecology and Obstetrics (FIGO) [20] and later converted to UICC-classification. In cases of missing stage-information and strong evidence of a metastatic disease (n = 23), these patients were staged as stage four.

### Completeness of therapy

Chemotherapy and radiotherapy were assessed with respect to completeness of the original intended treatment plan irrespective of reason for discontinuation. Local, simplified oncological therapy guidelines were: breast cancer stages 2–4 chemotherapy, stage 1 chemotherapy in case of high risk features and stages 3–4 additional radiotherapy; cervical cancer stages 2–4 concurrent radio-chemotherapy; non-Hodgkin`s lymphoma stages 2–4 chemotherapy; colon cancer stages 3–4 chemotherapy; lung cancer stages 2–4 chemotherapy; ovarian cancer stages 2–4 chemotherapy; head-and-neck cancer stages 2–4 concurrent radio-chemotherapy; and rectal cancer stages 2–4 concurrent radio-chemotherapy.

Complete chemotherapy was defined when patients received ≥85% of the intended cycles referring to a study showing a better 20 year relapse-free survival in breast cancer patients (52.3% compared to 31.5% relapse-free survival) [21]. We applied the same cut-off for completeness of radiotherapy. Local therapy plans differed from high-income countries due to lack of 3D radiation and limitations of the Cobalt-60 tele machine. Analysis on completeness of therapy was done for the five and four most common cancer types, wherever chemotherapy or radiotherapy applied.

### Time to therapy

Time to therapy was calculated between date of therapy planning and initiation of therapy. Patients with unknown starting as well as ending date of therapy were not included (n = 67 chemotherapy and n = 50 radiotherapy). Patients booked for palliative hemostatic-radiotherapy were excluded, because they received an immediate emergency-radiation (e.g. massive cervical cancer bleeding). Furthermore, patients receiving radio-chemotherapy were excluded from analysis of waiting time for chemotherapy because time to treatment mainly depended on radiotherapy.

### Statistical methods

Analysis was performed using SPSS Statistics, Version 23 (IBM Corp. Released 2015. IBM SPSS Statistics for Windows, Version 23.0. Armonk, NY: IBM Corp.). We obtained ethical approval (124/10/IM) from the Addis Ababa Medical Faculty Review Board and the Martin-Luther-University Halle Review Board. All data/samples were fully anonymized before accessed.

## Results

A total of 588 patient files were analyzed. To assure a representative sample, frequency of cancer entities within those files retrieved were compared with those registered in AACCR (n = 1149). This comparison showed a similar distribution which supported our assumption of missing files at random. The 10 most common cancer types are described in Table 1.

**Table 1:** Clinical and pathological characteristics of the study population (subgroup of AACCR*) compared to the AACCR cohort.

		Study population Number [n]	Study population Proportion [%]	Number in AACCR [n]	Proportion in AACCR [%]
Total population		588	100	1149	100
Age (years)					
<30		83	14.1	182	15.8
30–39		113	19.2	210	18.3
40–49		131	22.3	226	19.7
50–59		113	19.2	229	19.9
60–69		86	14.6	177	15.4
≥70		62	10.6	125	10.9
Sex					
Female		404	68.7	764	66.5
Male		184	31.3	385	33.5
Type of hospital					
Governmental		381	64.8	730	63.5
Private		207	35.2	419	36.5
Cancer entity	ICD-10 Code				
Breast**	C50-X	165	28.1	244	21.2
Cervix	C53-X	51	8.7	117	10.2
Colorectal	C18-X-C20-X	45	7.7	79	6.9
Non-Hodgkin-lymphoma	C83-X	40	6.8	66	5.7
Lung	C34-X	28	4.8	26	2.3
Sarcoma	C49-X	26	4.4	42	3.7
Thyroid	C73-X	22	3.7	48	4.2
Ovary	C48-X	18	3.1	43	3.7
Cancer of unknown primary	C80-X	15	2.6	27	2.3
Esophagus	C15-X	15	2.6	31	2.7
Others	/	163	27.5	426	37.1

*AACCR: Addis Ababa City Cancer Registry.

**breast cancer in male [n = 8].

The majority of patients (74.8%) were under the age of 60 years. More than two thirds (68.7%) were female. The largest group of patients with known performance status (24.1%) was lightly restricted by their disease (ECOG1). A high percentage of patients presented with a late stage 4 disease (38.8%) and a negligible proportion of cancer entities (2.0%) presented with stage 1. (See Table 2)

**Table 2:** Patients characteristics and treatment received in the study cohort.

	Number [n]	Proportion [%]
ECOG* at time of presentation		
Fully active (ECOG 0)	20	3.4
Lightly restricted (ECOG1)	142	24.2
Unable to work (ECOG 2)	89	15.1
Limited self-care, >50% in bed (ECOG 3)	50	8.5
No self-care, bed bound (ECOG 4)	11	1.9
unknown ECOG	276	46.9
Stage at time of presentation		
Stage 1	12	2.0
Stage 2	58	9.9
Stage 3	75	12.7
Unknown, probably stage 2 or 3	215	36.6
Stage 4	228	38.8
Any therapy received (operation, chemotherapy, radiotherapy)		
yes	475	80.8
no	113	19.2
Operation received		
yes	306	52.0
no	282	48.0
Chemotherapy for patients in demand (top 5 cancer-entities)		
yes	187	64.0
no	84	28.8
unknown	21	7.2
Worst case scenario chemotherapy (top 5 cancer-entities)		
yes	187	32.0
no	397	68.0
Radiotherapy for patients in demand (top 4 cancer-entities)		
yes	50	26.6
no	138	73.4
Worst case scenario radiotherapy (top 4 cancer-entities)		
yes	50	13.3
no	326	86.7

*ECOG = Eastern Cooperative Oncology Group.

About two thirds (64.8%) of patients received their therapy in a governmental hospital. One fifth of the patient cohort never received any operation, chemotherapy or radiotherapy; the majority (80.8%) received at least one therapeutic modality. One half (52%) of patients were operated for their primary tumor, and 54.1% of the whole patients cohort were treated with chemotherapy. One third (31.6%) of stage 4 cancer patients received a WHO-pain-ladder 3 medication.

As a worst case scenario, we assumed that no therapy was given to those patients whose file could not be traced. This estimated that 68.0% of the original AACCR cohort eligible for chemotherapy was not treated and 86.7% of the cohort eligible for radiotherapy was not treated.

### Completeness of chemotherapy for eligible patients

**Fig 1:**
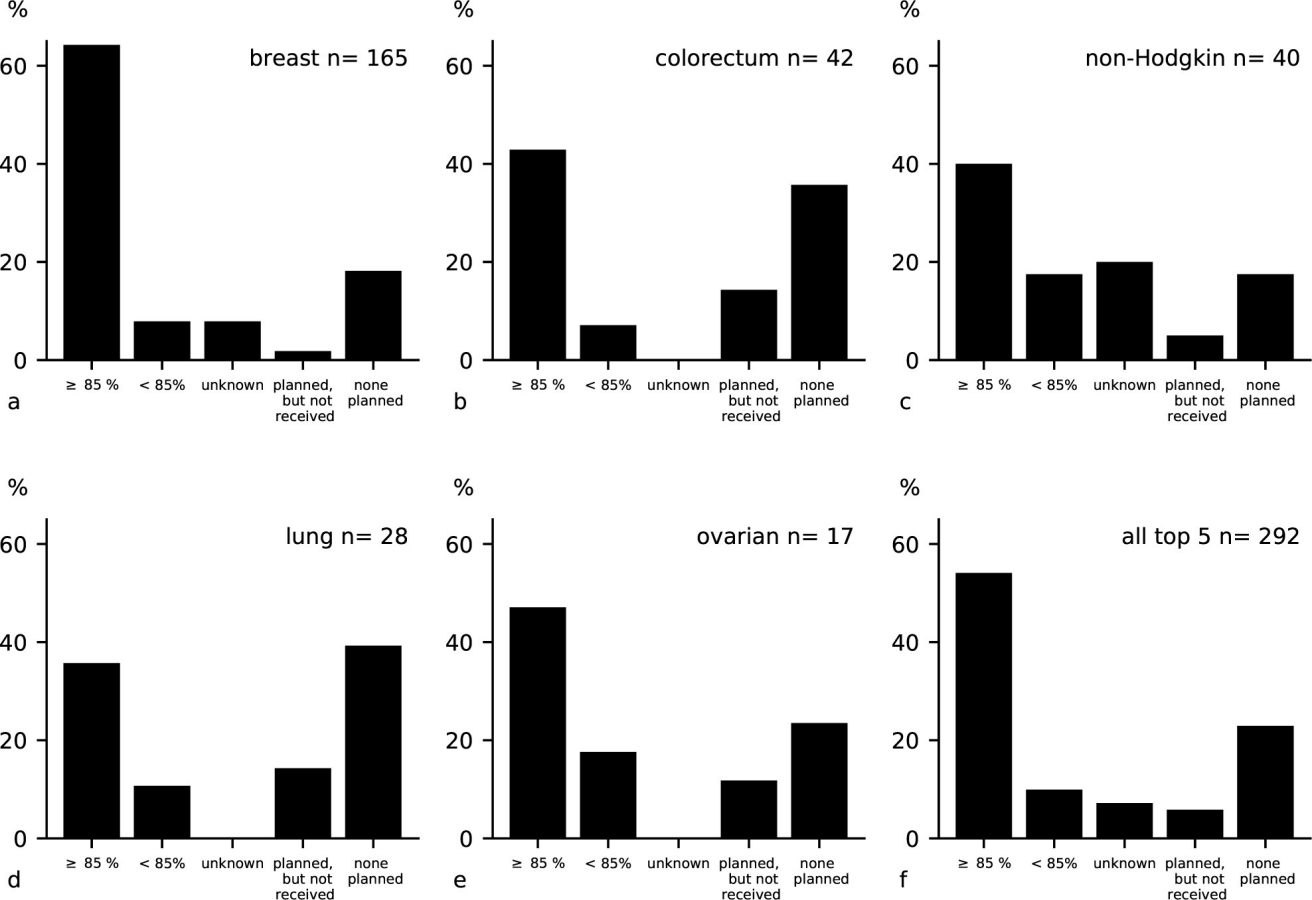
Completeness of chemotherapy according to local guidelines in top 5 cancer entities: (a) breast cancer stages 1–4; (b) colorectal cancer stages 3–4; (c) Non-Hodgkin`s lymphoma stages 2–4; (d) lung cancer stages 2–4; (e) ovarian cancer stages 2–4; (f) all 5 cancer entities together.

There were 292 patients (49.7%) in our study-cohort who had one of the five most common cancer types treated with chemotherapy according to local guidelines: breast cancer (n = 165), colorectal cancer (n = 42), non-Hodgkin`s lymphoma (n = 40), lung cancer (n = 28) and ovarian cancer (n = 17). Half of these patients (54.1%/ n = 158) received complete therapy. Breast cancer patients most commonly completed chemotherapy (64.2% of all breast cancer cases n = 106). Once chemotherapy was started 9.9% (n = 29) of all patients did not receive complete treatment. This could be due to progression of the disease, side-effects, economic, logistic or other reasons (personal information). A minority (5.8%/ n = 17) of all eligible patients was booked for chemotherapy, but eventually did not start. The largest proportion of them suffered from cancer of the lung (14.3%/ n = 4) and colorectal cancer (14.3%/ n = 6). One quarter (22.9%/ n = 67) of the patients eligible had no planned chemotherapy. The largest proportion of these were among lung (39.3%/ n = 11),colorectal (35.7%/ n = 15) and ovarian cancer patients (23.5%/ n = 4) and smallest in breast cancer (18.2%/ n = 30) and non-Hodgkin`s lymphoma (17.5%/ n = 7). 7.2% (n = 21) of patients had an unknown therapy status [Fig 1].

### Completeness of radiotherapy

**Fig 2:**
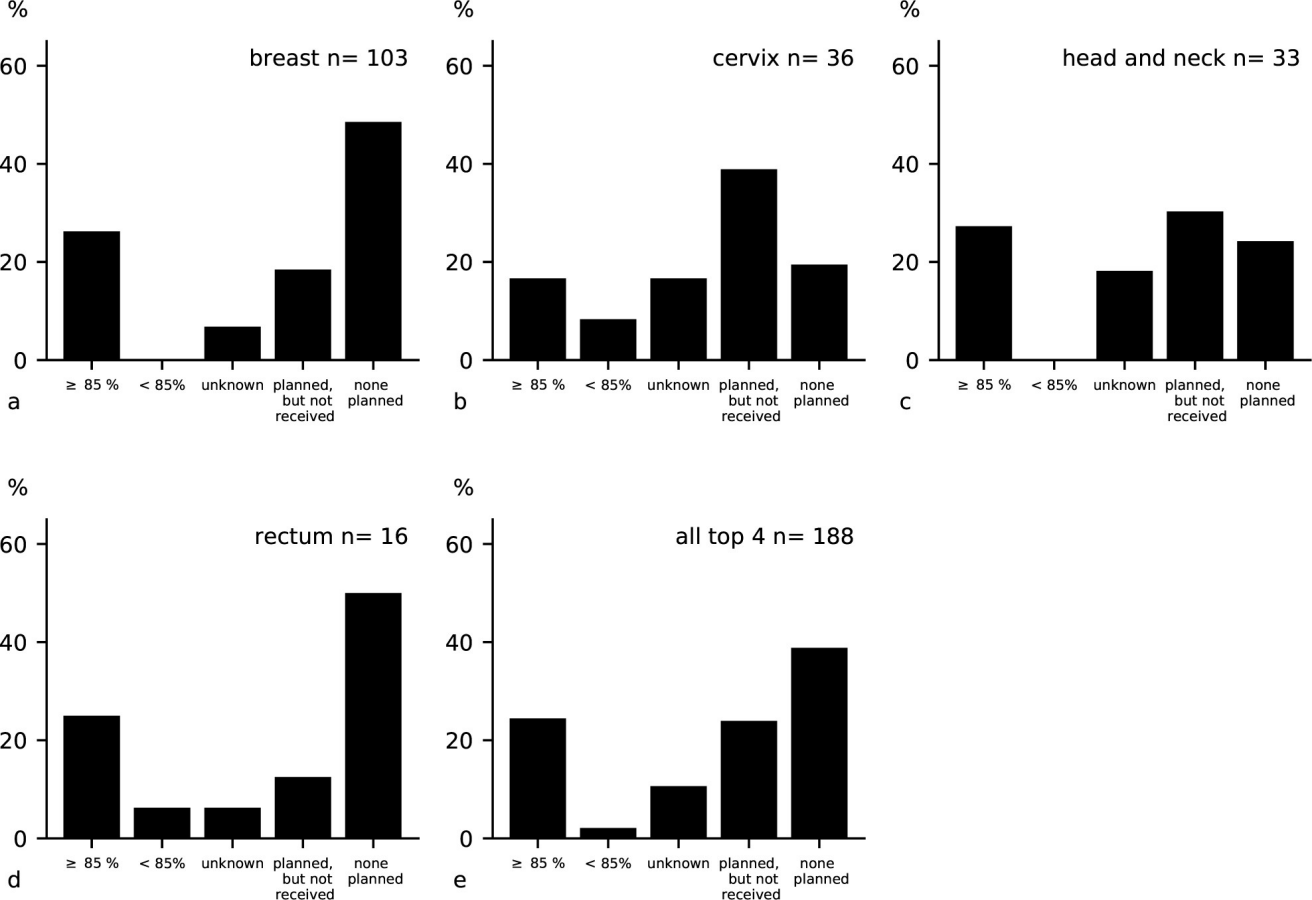
Completeness of radiotherapy according to local guidelines in top 4 cancer entities: (a) breast cancer stages 3 and 4; (b) cervical cancer stages 2–4 without single-shot; (c) head and neck cancer stages 2–4; (d) rectal cancer stages 2–4; (e) all 4 cancer entities together.

We found 188 patients (32.0% of the patients cohort) eligible for radiotherapy, of which breast cancer patients stages 3 and 4 were the majority (n = 103), followed by cervical stages 2–4 (n = 36), head-and-neck stages 3 and 4 (n = 33), and rectal cancer stages 2–4 (n = 16). One fourth (24.5%, n = 46) of these patients completed their prescribed dose of radiotherapy, with the proportions almost equally distributed among the entities. A very small patient group (2.1%, n = 4) received an incomplete radiotherapy of <85% of fractions, whereas a high percentage of patients (23.9%, n = 45) never started the planned radiotherapy. Radiotherapy was not prescribed for a high proportion of patients (38.8%, n = 73), despite their registration and eligibility [Fig 2].

### Waiting time

**Fig 3:**
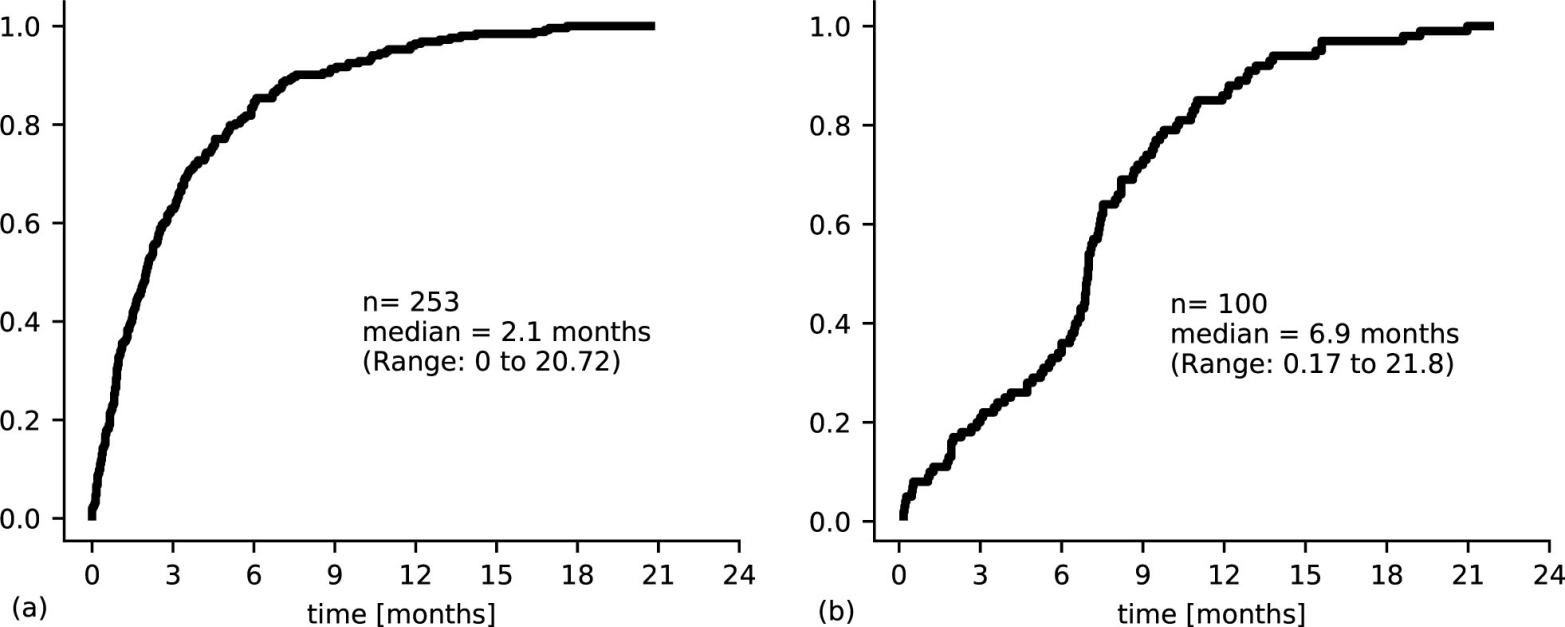
Cumulative probability of receiving chemotherapy (a) and radiotherapy (b) over time [in months].

The median waiting time for the 253 chemotherapy patients was 2.1 months (Range: 0 to 20.72). Out of 100 radiotherapy/radiochemotherapy patients eligible we found a median waiting time of 6.9 months (Range: 0.17 to 21.8) [Fig 3].

The median waiting time until the start of any of those two therapy options was 2.2 months (Range: 0–20.72). The majority (n = 253) had chemotherapy as their primary treatment and a small proportion (n = 30) received radiotherapy only.

## Discussion

In Addis Ababa about half of the eligible cancer patients received the planned chemotherapy within 6 months after diagnosis and about 25% received the planned radiotherapy within 12 months after diagnosis. Half of the patient cohort received oncologic surgery for their primary cancer.

We found a very young patient cohort mainly below the age of 60, consistent with the population structure of Ethiopia [22]. A high proportion presented with stage 4 diseases (38.8%) comparable with recent studies from Ethiopia showing 34.2% of cervical cancer patients [23] and 57–70.8% of breast cancer paients [24,25] presenting in advanced stages. A low level of cancer awareness, lack of screening programs [26], limited access to health-care institutions, poor financial situations and traditional medicine are often cited explanations in Africa [27], [28], [29]. Since pain is the most prevalent symptom in advanced cancer patients [30], we highlight an unmet need of treatment finding only one third receiving an adequate WHO-pain-ladder 3 medication [31].

Our findings of 2.2 months waiting time until the start of cancer therapy (essentially consisting of the time to chemotherapy) seems high but can still be compared to studies performed in other African settings: e.g. a 5 week median waiting time to cancer therapy in Ghana [32], 21.5 days to cancer therapy in Kenya [33], 1.3 months for breast cancer patients in Mali [34] and even 3 months to cancer therapy for patients in Botswana [35]. In contrast, patients in Germany wait 15 days until the start of cancer treatment [36].

A long waiting time for therapy and perceived inefficiency cause many patients to opt for alternative medicine in Ethiopia. Estimates suggest that more than 80% of health problems are treated by traditional health care practices, with cancer being among the top ten reasons [37].

Completeness of chemotherapy in our cohort was influenced by cancer stage and type. Early cancer stages 1 and 2 received complete chemotherapy according to guidelines (82.2%) more often than stage 3 cancer patients (67.3%). Breast cancer patients and non-Hodgkin`s lymphoma patients also received adequate therapy more often. Out-patient service without competition for beds had been installed for these patients requiring chemotherapy only. In contrast, chemotherapy for ovarian, lung, and colorectal cancer patients was only given to in-patients. The low bed-capacity probably reduced the chance of getting chemotherapy on time and is reflected by the comparably higher amount of patients left without therapy (48.3%).

A study from 2007 found rates of 50–60% of cancer therapy discontinuation in children in low-income countries, constituting a major cause of therapeutic failure [38]. Although these statistics relate to childhood cancer, our findings are similar and underline the need for improvement in cancer care.

In this population-based cohort we found a very long waiting time for radiotherapy of 6.9 months for patients eligible and a large portion of patients (23.9%) who never received their planned therapy. Similarly, a hospital cohort-study from Ethiopia mentioned the considerable amount of cervical cancer patients, who died while waiting for therapy and those patients, who outlasted their waiting time with significantly increased cancer stages. Proportions of advanced FIGO-stages over a time period of 2 months increased from 44.2% to 68.3% [23]. Such stage-migration would probably be even worse in our study with a median waiting time of around 6.9 months, showing a unique result due to lack of data from other African countries. Requirements for radiotherapy are considered much higher in low-income countries due to late stage presentation and thus, more commonly used palliative therapy concepts including radiotherapy [39]. A recent IAEA study showed a median unmet need for radiation in developing countries of 47% [40]. Having only one single Cobalt-60 machine in use for the whole of Ethiopia, waiting time will continue to increase due to the increasing patient load and prolonged radiation-times resulting from the decreasing efficacy of the machine.

### Outlook

This study shows the challenges Ethiopia is facing in the fight against cancer by looking more closely at population-based provision of cancer therapy. Despite the deficits, steps have been taken to improve the situation. A new oncology program for nurses was established in 2015, the first oncology residents have completed their 4-year training in 2017 and a new oncology outpatient center opened at the beginning of 2016. A national cancer control program has been approved and cervical cancer screening has started. Moreover, new radiotherapy-machines were ordered. These changes are hopeful and demonstrate the increased cancer awareness of politicians and policy makers. At the moment, however, it is impossible to serve the demand for cancer care in Ethiopia.

### Limitations

This study has some limitations. Despite our population-based approach, files could only be retrieved for about half of the patients selected from the registry. We assume those without files retrieved have likely not received any therapy. Information about patients with early deaths might also be underrepresented. Therefore, our cohort tends to represent the best treated patients in the country and population-based access to cancer therapy is probably lower. Furthermore, being a retrospective study, some information might have been misinterpreted due to incomprehensible documentation. We assume this is at random. We grouped patients in need of a specific therapy according to general guidelines as the basis to analyze the completeness of therapy. We were unable to account for individualized therapy approaches in stage four patients, any non-standard therapy could have falsely been classified as not complete. Personal therapy recommendations by the physician or individual reasons of the patient not to plan access to such guidelines could not be taken into account due to inconsistent documentation. Therefore, patients classified as „not received“, despite guideline recommendation may well have had their own reasons not to receive therapy. Besides, therapy might have been delayed by patients themselves due to individual reasons and have lead to longer waiting times.

## Conclusion

In this study, we present completeness of cancer therapy and waiting time as documented from 588 out of 1149 patients of the only population-based cancer registry in Ethiopia. Our findings that only half of those patients received adequate chemotherapy and one fourth received adequate radiation underscores the need for system-wide measures to improve delivery of cancer care. We were unable to obtain detailed reasons for non-adherence to therapy- whether this was a problem on the health care provider`s, on patients`or on the logistics side. The known lack of staff, chemotherapy and radiotherapy capacities strongly points towards a critical shortage of health care provision rather than patient decisions against therapy. We also saw that waiting time for chemotherapy was relatively short (2.1 months) compared to waiting time of 6.9 months for radiotherapy, which clearly shows the need for additional radiotherapy facilities. Once therapy was started, the drop-out rate for both therapies was relatively low (definite 9.9% for chemo- and 2.1% for radiotherapy) which points towards good patient adherence and service delivery. The results of this population-based study show the tremendous challenges Ethiopia is facing in the fight against cancer with need for expansion of existing structures to improve access to timely, cost-effective and high-quality care [41].

## Supporting information

S1 Table Addis ababa cancer registry data. (XLSX)
